# Over the Garden Wall

**Published:** 1884-03

**Authors:** C. M. Wright

**Affiliations:** Cincinnati, Ohio


### "OVER THE GARDEN WALL."

BY C. M. WRIGHT, CINCINNATI, OHIO.

### IS DENTAL CARIES A DISEASE?

If this is a simple question, there should be but one answer to it, and that answer should be so positive and so clear that every intelligent dentist could answer it promptly. There should be no doubt cast about the answer. It should be n, o, No; or y, e, s, YES, in capital letters. We have been calling it a disease for many years. Old text-books have classed it among diseases of the teeth (*vide* Hunter, Lefoulon, Bond, Harris, Taft, etc.). Later text-books on dental *pathology* treat of dental caries. An immense amount of scientific work has been done on its etiology. Prizes are offered! for the best essays on the subject. The youngest students in dental colleges enroll themselves under the banner of one or the other theory about the etiology of dental caries. We have battles among journalists and disputations innumerable about the cause of this important thing. Is it a disease at all? Here in Ohio we are just as much interested in the subject, and know as little about it as many dentists in other sections of our beloved country, and we therefore discuss it warmly at every meeting of every society in the grand old State. "The Etiology of Dental Caries" is as regularly served as one of the important dishes at our *table d' hote;* as soup, fish, or the roast is at any banquet *a la mode.*

At the regular meeting of the Mississippi Valley Dental Association, March, 1883, in discussing the second subject of the programme, viz.: "Etiology and Pathology of Dental Caries," Professor J. Taft is reported as saying: "Decay of the teeth is not a pathological process. * * * The statement that dental decay is a pathological process is misleading, and should be corrected." In reply to a direct question as to whether he regarded dental decay as a *disease* or not, he replied, "I do not; of course, caries is preceded by disease. As the process goes on there is a line of demarkation between dead and living. In the dead part there can be no disease." Again: "In the incipiency of dental decay pathological processes take place, but not after devitalization."

At the meeting of the Ohio State Dental Society, October, 1883, in the discussion of the usual topic, "Etiology of Dental Caries," Dr. Rehwinkel answered the question—"do you recognize dental decay as a pathological condition?" in this way: "Well, if it be not so at its incipiency, it is very apt to soon become so." Drs. Taft and Rehwinkel are as well known and as highly esteemed by the readers of the INDEPENDENT PRACTITIONER as they are by the entire dental world of this age, and they have discussed publicly this question of the etiology of dental caries as often and as intelligently as any two men in any other State. They are authorities in the West, and yet, after reading their opinions, expressed in 1883, in regard to whether *caries dentium* is a disease or not, who can answer on authority, yes or no? That is, who can say whether it is a disease just in the beginning, or just after a while? Who can say exactly when it is a disease, or exactly when it is not?

Dr. Atkinson clearly defines the term *decay*, and gives its Latin origin: *de*, down, and *cado*, I fall; and in the September number of the INDEPENDENT PRACTITIONER, page 475, is reported as saying: "All this trouble about understanding what decay is has come from invariably attributing it to the seat of the activity, rather than the activity itself, and if it comes down to saying whether we really know what decay is, or that we do not know, *it depends upon our definition of the term decay.*"

Are dental societies always looking for the cause of something when they don't know what that something is? Would it not be wise to find out *what* it is before they try to find out *why* it is? or, at least, does it not seem reasonable that we should have a definition that is clear to two men in the same State, as a preliminary to discussion?

Physiologists and pathologists have had considerable trouble in getting down to clear definitions of *health* and *disease.* They have disputed about the line of demarkation between them, and seem to have no sharply defined ideas on the question of when physiology leaves off, and pathology begins. They have, within a few years, shifted the line from one side to the other of certain hypertrophies. Dentists who are constantly cutting into and having under observation one class of tissues, seem to have no positive knowledge about an exact line between healthy and diseased tooth tissue. Here is a tooth, the physical properties of which are so good that every one would pronounce it healthy, and yet its elements may be at this moment undergoing a deterioration, a metamorphosis from impaired nutrition, and it may be slowly but surely getting into a condition that "predisposes" to decay, or to the disease, dental caries. We cannot see the deviation from the perfect type that starts that tooth toward the pathological condition. This is excusable. We may never know all the laws nor the influences that affect the tooth in its evolution from an embryonic layer of a germ, nor the influences for evil that preceded its beginning in that layer, but it seems to me that there should be no dispute about the coarser differences that distinguish a decayed tooth from one not decayed. There should be no indistinctness in our appreciation of conditions and terms in these later differentiations of tooth tissue. The line of demarkation should not move along the cavity, as Prof. Taft claims, nor should there be doubt, as Dr. Rehwinkel expresses, about the beginning or the ending of a process in so simple a question as the one asked in 1883, in Ohio.

The question should be answered, the line defined, and the name agreed upon in 1884. Is not the falling down of the elements of a tissue, as we *see* it in enamel, dentine, or cementum, disease? Are not *tissues of the body* that are in process of tumbling together from any cause, in a pathological state? When acids (organic or chemical) effect chemical changes in enamel, are there not anatomical changes produced in the tissue? Can these anatomical changes in living tissue (or in the non-living formed material that has once been bioplasm)—this falling down of the elements of tissue that we call decay or caries—be in accordance with health, or the normal type, or can it be compared with any physiological process for the disposal of waste formed material?

In short, cannot this whole process be distinctly described as pathological, and as "over the garden wall," that lies between physiological and pathological estates? When the process, with its accompanying possibilities and probabilities of micro-organisms, hyper-sensitiveness, pulp activity, nutritive excitation, etc., etc., extends into dentine, are we not even then over the wall, or are we carrying it along with us? I want to be sure about this. It is pleasant enough to sit on the fence in the moonlight, or in the shade on a hot day; but in utter darkness, not knowing what lies on either side, it is an uncomfortable situation. I am perfectly aware of some of the questions that might arise from Dr. Taft's view of the process. "A dead man is not in a pathological condition," strictly speaking. He has passed beyond the physiological and pathological states, perhaps. There is also a falling down or decay of dead teeth, as witnessed in the crowns of natural teeth set upon pivots and plates, and subjected to the same influences that surround and destroy living tissues in the oral cavity. There are also other complicated processes in other living tissues, which lead to the arrest of the circulation, to local death, to mortification, etc., such as the varieties of gangrene. Decomposition will take place in muscle in a butcher shop, but if mortification take place in any part of the butcher's body, although the processes may be similar, one may be called a deviation from a state of health, and in the province of pathology, while the other does not get anywhere near to the "line of demarkation" between healthy and diseased tissue. Our question needs settling. Is *caries dentium* a disease, or is it simply the result of disease? and what is the disease that causes caries?

